# Between East and West: Unitarian Service Committee’s medical mission in post-World War II Poland

**DOI:** 10.1017/mdh.2026.10072

**Published:** 2026-10

**Authors:** Kacper Rosner-Leszczyński

**Affiliations:** Institute of History, University of Wrocław, Wrocław, Poland

**Keywords:** Unitarian Service Committee, Aid, Medical mission, Poland, World War II, Public health

## Abstract

In the aftermath of the Second World War, Poland faced immense public health challenges amidst widespread destruction and political transformation. This article explores the largely overlooked medical mission of the Unitarian Service Committee (USC) in Poland, situating it within both humanitarian and geopolitical contexts. Drawing on archival research and press accounts, the study reconstructs the USC’s multifaceted aid efforts, including its Medical Teaching Mission, the establishment of the Piekary Śląskie hospital, and tuberculosis control initiatives. The analysis highlights how the USC combined direct medical assistance with educational outreach, aiming to support long-term recovery. However, operating behind the Iron Curtain, the Committee’s activities were subject to both surveillance and propaganda, revealing the entanglement of health and politics in Cold War Europe. This case offers new insights into the role of non-governmental actors in shaping postwar public health and diplomacy in Eastern Europe.

## Introduction

In the immediate aftermath of the Second World War, war-torn Europe faced not only the monumental task of political reconstruction but also the dire need for humanitarian and medical relief. Among the organizations that responded to this call, the Unitarian Service Committee (USC) played a particularly significant role through its international relief efforts. While much scholarly attention has been devoted to the USC’s activities in countries such as Greece, Italy, France, and Japan, the organization’s work in Poland remains understudied. This study seeks to fill that gap by examining the USC’s medical mission in this country – devastated by occupation, conflict, and the shifting postwar order. Set against the backdrop of the emerging East–West conflict, the USC’s presence in Poland became politically charged. As tensions deepened between the Soviet Union and the United States, even medical relief work was viewed with suspicion, caught in the crosscurrents of ideological control.

Born out of the geopolitical urgency of May 1940, the USC was established in Boston, USA with the specific mandate of aiding victims of Nazi aggression. The USC carved out a distinct identity by rejecting political neutrality and openly aligning itself with the Allied struggle against fascism.^1^ As Jon Arrizabalaga and Àlvar Martínez-Vidal rightly noted: ‘Soon after the Nazis invaded France and occupied Paris, the USC established its first European office in Lisbon in order to aid refugees in their transit to the Americas across the Pyrenees and Spain. Lisbon was primarily chosen for geostrategic reasons, as the capital of a neutral country Portugal, and one of the few important non-military harbours in continental Europe that was still in operation.’^2^ USC was sponsored by Unitarian churches in the United States and supported by contributions from the American people through the National War Fund.^3^ The National War Fund ceased to exist on 31 December 1946, after which the USC faced the challenge of raising funds through Unitarian churches and other organizations and individuals.^4^

As a religiously affiliated organization, the USC maintained only limited and indirect ties with the U.S. government. Although it operated offices in Boston, its activities cannot be understood as the result of close or formal collaboration with American state authorities.^5^ In missions solely sponsored by the Unitarian Service Committee, most personnel were American, with non-American scientists drawn upon only in exceptional instances. In missions jointly sponsored by the WHO and the USC, approximately half of the professional staff were from countries other than the United States, but the Chairman was always American.^6^ The philosophy of the organization, which sought to distance itself from treating aid as a weapon of the Cold War, is perfectly captured in an excerpt from a document significantly titled ‘USC Medical Missions Demonstrate Democracy in Action’:

The medical program goes directly to a highly important segment of the population. Once the approval in principle of a foreign government is obtained, the negotiations, arrangements, and program are worked out directly with the people concerned and not through diplomatic channels. The program is entirely separated from those complexities of international politics which cannot fail to affect government aid in these days when assistance and reconstruction is frankly admitted to be a political weapon in the cold war.^7^

By framing direct medical assistance as a form of ‘democracy in action,’ the Committee sought to bypass the paralysis of diplomatic protocols and insulate its humanitarian work from the ideological weaponization of aid that defined the early Cold War. In the context of this study, diplomacy was not understood through the traditional lens of formal state-to-state relations, but rather as humanitarian diplomacy. This referred to the strategic ability of a non-governmental organization (NGO) to negotiate its presence and operate across hostile political boundaries. Humanitarian diplomacy, as defined by Olga Bogatyreva, is a complex process of interaction between various actors, e.g. NGOs, aimed at mobilizing resources and political support to alleviate human suffering and ensure the rights of those in need.^8^ In this study, this definition was applied to the USC. By framing its aid as ‘democracy in action’ and apolitical work, the USC navigated the restrictive environment behind the Iron Curtain.

Early insights into the USC’s medical relief work in postwar Europe were provided by Chester M. Jones, a field physician whose first-hand account, ‘Medical Mission to Greece and Italy’ (1949),^9^ remains a foundational narrative of the organization’s operations in Southern Europe. His observations highlighted the logistical challenges and medical priorities in the early phase of USC missions, setting a precedent for subsequent humanitarian deployments.

In a separate body of scholarship, Susan Subak and William F. Schultz,^10^ Haim Genizi,^11^ Aurelio Velázquez-Hernández,^12^ Jon Arrizabalaga and Àlvar Martínez-Vidal^13^ have explored the USC’s efforts on behalf of refugees and displaced populations during and after the war. Genizi’s ‘Christian Charity’ details the USC’s relief activities for refugees from Nazism, emphasizing its moral motivations and the practical assistance it provided in Europe. Velázquez-Hernández, focusing on the USC’s Marseille office, investigates the organization’s role in the American aid networks that supported Spanish Republican refugees, shedding light on its political and logistical strategies in wartime France. Together, their work situates the USC not only as a medical actor but also as a humanitarian agency engaged in the broader refugee crisis of the 1940s.

Another important strand of scholarship comes from Shigemasa Ikeda^14^ and Akitomo Matsuki,^15^ who have studied the USC’s medical mission to Japan, particularly its contributions to the field of anaesthesiology. Ikeda documented how the USC facilitated knowledge transfer between American and Japanese physicians in the early 1950s, focusing on anaesthetic practices. Matsuki further explored this theme by examining the influence of Dr. Saklad’s lectures during the first USC mission to Japan. These studies underscored the technical and educational dimensions of the USC’s postwar missions, illustrating how American medical personnel played key roles in reconstructing medical systems in devastated countries. This topic has also been addressed in other works, including a more extensive study of the margins, e.g. by Miklós Dániel^16^ or Hans Herken.^17^

While these accounts have significantly broadened our understanding of the USC’s international scope, Poland remains largely absent from the historical narrative. Although the historiography on postwar relief in Poland includes significant work on the activities of the United Nations Relief and Rehabilitation Administration (UNRRA),^18^ scholarly attention to non-governmental and religious-based aid organizations such as the USC has been limited. Studies on UNRRA have shed light on the scope of international assistance, the logistical challenges of aid distribution, and the political tensions surrounding foreign involvement in early communist Poland. However, these works tend to emphasize state-level cooperation and overlook the role of smaller, more ideologically diverse actors.

This article examines the medical mission of the USC in postwar Poland, situating it at the intersection of faith-based humanitarianism and Cold War politics. It is crucial to clarify that the USC’s engagement was not a singular event, but a multi-stage program spanning from the initial 1945 assessment through the 1946 Medical Teaching Mission to long-term hospital projects in Piekary Śląskie and elsewhere. The central argument posits that while the USC operated as a non-governmental, religious organisation driven by the ethos of ‘democracy in action’ and while it explicitly avoided political involvement, its presence behind the Iron Curtain was inevitably politicised by the local context. This paradox was best exemplified by the phenomenon of the Piekary Śląskie hospital, where humanitarian staff worked under the constant, intrusive surveillance of the Polish Security Office, treated simultaneously as vital allies in reconstruction and potential intelligence threats. The article analyses how, despite this atmosphere of suspicion, the mission functioned as a ‘porous membrane’ facilitating professional exchange and knowledge transfer (a ‘Nylon Curtain’^19^), even as it was manipulated by the communist regime’s propaganda machinery aiming to reconcile the need for Western aid with anti-Western ideology. The USC’s medical mission in Poland is analysed here not merely as a relief effort, but as an initiative that fostered a transnational epistemic community. This term refers to a network of professionals, in this case, American and Polish physicians, who shared a commitment to a specific domain of knowledge.

To provide a comprehensive reconstruction of the USC’s activities in postwar Poland, this study relies on the triangulation of diverse primary sources. The core empirical material is drawn from the USC archives (held at the Andover-Harvard Theological Library), including official mission reports, administrative correspondence, and financial records. These institutional documents are complemented by ego-documents, most notably the diary of Dr. Wade Oliver, a participant in the 1946 Medical Teaching Mission, whose personal reflections offer a vivid, first-hand account of the humanitarian landscape and the scale of destruction encountered by American physicians. Furthermore, to capture the complex geopolitical context of the mission, the analysis incorporates Polish press accounts from the period, found in journals such as *‘Dziennik Zachodni’* and ‘*Głos Wielkopolski’* which illustrate how the mission was presented for propaganda purposes. The selection of these specific titles is grounded in the geography of the USC’s operations: *Dziennik Zachodni* served the industrial Silesian region, home to the flagship hospital project in Piekary Śląskie, while *Głos Wielkopolski* covered the Poznań area, one of the key locations for the lectures delivered during the Medical Teaching Mission. Unlike the central party organs, these regional journals were tasked with addressing local concerns, including health crises and reconstruction, making them the primary medium through which the average citizen encountered information about foreign aid. However, it is crucial to note that by 1946–1947, these publications were fully integrated into the state’s apparatus of control; they were subject to strict censorship and operated within guidelines set by the communist authorities. Consequently, the narrative found in these pages was not an independent journalistic record but a carefully curated product of state propaganda. The decision to use *Głos Wielkopolski* and *Dziennik Zachodni* as a primary case study allows for a focused analysis of the regime’s ‘dual narrative’ strategy: a calculated effort to legitimise the government by showcasing tangible relief (the provision of penicillin, equipment) while simultaneously engaging in broader anti-Western rhetoric to align with Soviet geopolitical interests. The focus on the Polish regional press rather than purely official documentation stems from the fact that it was the media that served as the main instrument for building the public image of the mission and managing its social reception. Thanks to this approach, the study focuses not on the administrative history of the institution, but on humanitarian diplomacy with particular emphasis on communist propaganda about the mission and its description to citizens. The national press is also used as a supplementary source. Finally, the study utilises Kornelia Banaś’s work, which examined records from the communist security services regarding the surveillance of the hospital team in Piekary Śląskie, thereby revealing the political pressures and intelligence scrutiny under which the humanitarian staff operated. This multi-perspective approach allows for a critical assessment of the mission not merely as a medical intervention, but as a phenomenon situated at the intersection of humanitarianism and Cold War diplomacy.

## Aid to Poland

In the immediate aftermath of the Second World War, the Polish healthcare system was in a state of near-total collapse. Years of occupation, destruction, and human loss had devastated both the physical infrastructure and the personnel base of the country’s medical services.^20^ Thousands of physicians were killed during the occupation – many in Nazi concentration camps – and the Jewish community, which had included a large proportion of Poland’s highly trained medical professionals, was annihilated. Public health challenges were enormous. Epidemics of typhus, tuberculosis, and dysentery swept through displaced populations, while millions of returning refugees and repatriated labourers strained the already broken system. Rebuilding efforts were hampered by limited resources and by the political and logistical upheaval of the emerging communist regime. While the newly established Ministry of Health sought to reorganize and centralize healthcare provision, international aid – including from organizations like the USC – played a crucial role in addressing both immediate emergencies and longer-term reconstruction. Medical teaching, infrastructural repair, and the importation of vital supplies such as antibiotics, X-ray machines, and surgical tools were urgently needed to revive the system.

In this context of acute national crisis and systemic collapse, international humanitarian organisations emerged as essential partners in Poland’s recovery. Among them, the USC distinguished itself through its targeted focus on medical aid and professional education (but not only). Between 1944 and 1946, USC shipped nearly two million pounds of food, clothing, and medical supplies. Help was provided to France, Switzerland, Portugal, Netherlands, Great Britain, Austria, Hungary, Norway, Belgium, Palestine, Greece, Yugoslavia, Romania, Germany, Italy, Czechoslovakia, and Poland.^21^ In 1946, USC operated 13 medical projects, e.g. USC Innsbruck Dental Clinic, Hospital Varsovie in Toulouse, USC Sanitary Engineering Fellowships in Italy, the Italian Medical Nutrition Mission, and – the subject of this article – the Polish Medical Teaching Mission.^22^

After the Second World War, Poland received tremendous assistance from numerous organisations. For example, in 1948 the American Joint Distribution Committee served 93,000 meals each month through 59 canteens. Activities of these organisations encompassed various welfare services, focusing on child and infant care, medical services, anti-tuberculosis efforts, and cultural activities.^23^ The American Red Cross sent supplies included basic medical and hospital supplies, clothing, layettes, powdered milk, and vehicles.^24^ The Cooperative for American Remittances to Poland offered various standard packages, including food, clothing, and household items.^25^ Given the sheer number and variety of organisations involved, continuing with a detailed enumeration would be both redundant and impractical. Instead, it is more meaningful to focus on broader patterns of support and the strategic roles played by USC.

**Figure 1.**
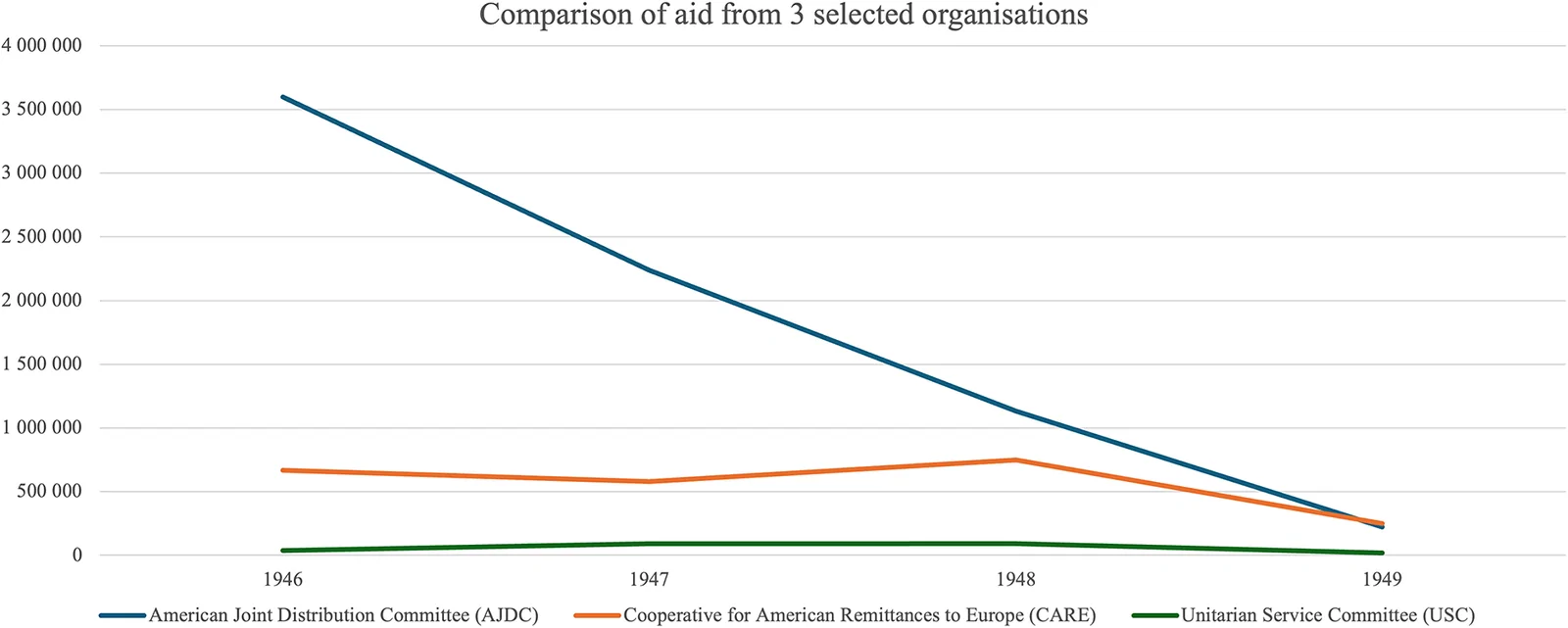
Comparison of aid from 3 selected organizations. Figure 1. long description. *Source*: AHTL, bMS 16103, Box 29, Folder 18, 93–102; Łaptos, *op. cit.* (note 18), 491.

According to estimates compiled by Józef Łaptos, which documents the financial value of humanitarian aid delivered by charitable organisations cooperating with UNRRA in postwar Poland, the USC provided a total of 243,634 USD in assistance between 1946 and 1949. Although this figure is modest in comparison to larger actors such as the American Joint Distribution Committee (over 7 million USD), the War Relief Services – National Catholic Welfare Conference (over 7 million USD), or the Swedish Relief Organisation (over 11 million USD), the USC’s contribution is notable in light of its specific medical focus and targeted engagement.^26^ The graph in Fig. 1 illustrates the trends in aid provided by three selected organizations – AJDC, CARE, and USC – to Poland between 1946 and 1949. It shows a clear downward trend, with aid sharply decreasing each year, and visible inequality in offered aid.

Following the devastation of the Second World War, the USC gradually expanded its direct aid to Poland, shifting from earlier relief efforts focused primarily outside the country’s borders. During the war, USC provided crucial assistance to Polish refugees in Switzerland, unoccupied France, and Portugal. This aid included food, clothing, medical care, living stipends, and vocational training. Even after the war, the Committee continued to support a number of Polish cases in Western Europe – primarily elderly individuals or those in poor health who were unable to return to Poland.^27^

The USC provided aid for humanitarian reasons, focusing on medical relief and the advancement of medical science in Poland. The mission’s primary purpose was to bring the latest information about medical science to countries that had suffered from isolation and destruction of material facilities during the war years. It also aimed to facilitate an exchange of knowledge and create international friendships on a scientific level, minimising political and nationalistic friction. The USC’s work was described as non-controversial, with doctors focusing on professional work and helping to cure the sick and rehabilitate medical standards. The organisation consistently avoided involvement in politics, which contributed to its success and reputation.

The USC’s Polish Medico-Social Mission, beginning its work in July 1945, initially focused on providing medical and social aid in various camps across France (Brittany and the Aude department).^28^ The mission faced challenges due to the poor conditions in the camps, including inadequate sanitation, food shortages, and a lack of medical supplies. The team, composed of doctors specialising in various fields, nurses, and social workers, conducted medical examinations, provided treatment, and distributed supplies. They also worked to improve living conditions and morale in the camps by organising educational programs and community activities. By September 1945, the mission had examined 2350 people in various camps. In October 1945, the mission was summoned to Verdun to assist with the repatriation of 17,293 Polish deportees to Poland.^29^ The mission ensured the deportees travelled in acceptable conditions, providing food, medical care, and administrative support. Despite difficult conditions, including late-arriving and poorly organised convoys, the mission successfully facilitated the repatriation of approximately 18,000 deportees in 5 weeks, working in cooperation with the Polish Embassy, Polish Red Cross, and French authorities.^30^

A major initiative undertaken by the USC was the 1946 Medical Teaching Mission, when a group of eminent American physicians and scientists who travelled across Poland to share recent advances in medical science. The mission visited leading medical schools in Warsaw, Kraków, Lublin, Wrocław, Poznań, Łódź, and Gdańsk, offering lectures, demonstrations, and roundtable discussions. The doctors also donated substantial quantities of textbooks and medical materials to Polish institutions. Although formally directed by UNRRA due to the lack of a USC office in Poland at the time, the mission laid the groundwork for future USC educational and medical projects.^31^ One of the participating physicians, Dr. Wade Oliver from the Department of Bacteriology at Long Island College of Medicine, noted his personal impressions of postwar Poland in a diary that offers a vivid and deeply human account of the mission’s context. His reflections not only complemented the official narrative of the USC’s efforts but also underscored the immense medical and infrastructural challenges Poland faced at the time. In particular, Oliver’s description of Warsaw conveyed the scale of destruction and the emotional impact it had on those attempting to provide relief. His impressions of Poland were overwhelmingly shaped by the devastation he witnessed and the stark contrast between the suffering of the populace and the efforts of UNRRA to alleviate it. He repeatedly described Warsaw as a ‘City of the Damned,’ emphasising the ‘incredible physical demolition and ruin’ caused by the German occupation: ‘As I was soon to find out, Warsaw has only one doctor for each 3,600 people; 4,000 Jewish physicians, many finely trained, were wiped out. There was at least 70% hospital destruction in Warsaw, and it has been estimated that it would require 100 million dollars to put the hospitals of Warsaw back into first class condition. Incidentally, at least 90% of the buildings in Warsaw are utterly uninhabitable’.^32^ The scale of destruction was so profound that he felt like an archaeologist exploring an ancient, decayed city.^33^

Perhaps the most significant and enduring USC project in postwar Poland was the Hospital Team. Originally organized in Switzerland in 1944, this team of Polish doctors and social workers first operated in France and Germany, serving displaced persons by conducting medical surveys and assisting with repatriation. In 1946, after improving conditions at a UNRRA hospital in Wermelskirchen, Germany, the team moved to Poland under the leadership of Dorothea B. Jones, who was the American director of the original USC Polish Medical Team that arrived in Poland in November 1946 (USC Director for Poland). After consulting with Polish authorities, the hospital in Piekary Śląskie – located in an industrial region with high rates of mining injuries – was selected as the base for the Committee’s efforts. The hospital was already up and running before the outbreak of the Second World War but was severely damaged during that conflict. In 1946 the authorities decided to repair it and put it into operation. Due to the high costs, assistance was offered by the Polish National Health Insurance Agency (Zakład Ubezpieczeń Społecznych), UNRRA, and the USC. The Piekary hospital was intended not only as a treatment centre but as a model of modern medical care and education. With supplies shipped from abroad and medical staff trained in the latest American techniques, the USC aimed to reduce the long-term effects of mining accidents and to improve regional public health standards. The facility was also envisioned as a centre for medical training and demonstration, continuing the pedagogical goals of the earlier Medical Mission. The Committee anticipated a 3-year engagement with the hospital, with the goal of leaving behind lasting infrastructure, equipment, and professional development for the Polish medical community.^34^

Beyond institutional work, the USC also engaged in more general relief. For example, in December 1946, the Hospital Team distributed clothing packages to 800 impoverished children in Bytom. Further shipments of clothing, fur garments, shoes, and educational kits continued into 1947, with donations including 2,000 sets of dentures, 1,000 kilograms of vitamins, and baby food.^35^

According to estimates by the Supreme Extraordinary Commissariat for Combating Epidemics in Poland, the number of tuberculosis cases between 1945 and 1948 was approximately 1,200,000.^36^ In this aspect, too, Poland could count on the assistance of the USC. They undertook to provide the necessary X-ray machines,^37^ which were indispensable for TB detection tests.^38^ Their training was not only used by Polish doctors but also by patients who had the opportunity to undergo treatment at that time.^39^ At the same time, lectures were held in many Polish cities, including ‘Programs for Tuberculosis Control with Special Reference to Case Finding by X-Ray’ by Dr. W. Edward Chamberlain, Professor of Radiology at Temple University Medical School, or ‘Treatment of Pulmonary Tuberculosis by Temporary Collapse Therapy’ by Dr. Jean Good, Chief of Clinic of Thoracic Surgery in Basel, Switzerland.^40^ Moreover, in 1945, the USC sent a ton of DDT (Dichlorodiphenyltrichloroethane – synthetic insecticide) to the Polish Ministry of Health to help control the risk of epidemics. That same year, the Committee’s director conducted an exploratory mission to Poland to assess needs and potential relief initiatives. A symbolic shipment of food and clothing followed in 1946, and soon after, regular packages were sent to individuals in need throughout the country.^41^

Through its relief work in Poland, the Unitarian Service Committee combined humanitarian aid with educational and institutional support, aiming to contribute not only to immediate recovery but also to long-term national resilience. Its efforts illustrate the broader role of American humanitarian organisations in postwar Europe – particularly in supporting sectors such as public health, education, and social welfare amid the continent’s reconstruction.

## Between health and politics

The political context in which the USC operated was far from static; rather, it was defined by the fluid and gradually hardening realities of the early Cold War. The period between 1945 and 1948 constituted a specific era where the wartime alliance had fractured, but the geopolitical division of Europe had not yet fully ossified into the hermetic seal of the Iron Curtain. While Winston Churchill had famously deployed the metaphor in 1946, in practice, the boundaries of the Soviet sphere remained permeable to Western aid, driven by the sheer necessity of postwar reconstruction. As Sandrine Kott argued, internationalism did not cease immediately with the onset of political tensions; instead, diverse forms of trans-systemic circulation, particularly of technical expertise and resources, persisted across the emerging divide.^42^ It is important to note that the Truman Doctrine, widely regarded as the formal onset of the Cold War’s policy of containment, was not announced until March 1947. By contrast, the USC’s Medical Teaching Mission was planned and deployed in 1946. This chronological precedence is crucial, as it indicates that the Committee’s engagement originated in a fluid period, seizing a fleeting window of opportunity before the geopolitical fault lines fully calcified.

One of the most important things of concern to the communist authorities was the hospital in Piekary Śląskie. From 1947 onwards, the American specialists who came to the site became the subject of surveillance by the local Public Security Office.^43^ Between 1947 and 1950, the officer conducted a secret investigation under the code name ‘Szpital’ (‘Hospital’). One person who aroused suspicion among the communists was Dr. Janusz Draab (1918–1988), a Polish orthopaedic surgeon. What raised suspicion was his past association with national independence organisations, including the Home Army (Armia Krajowa, AK), a Polish underground resistance movement during the Second World War and considered enemies of the communists.^44^ It is also possible that his good knowledge of English and the fact that he had a car imported from abroad attracted the authorities’ attention. However, there was no evidence to support any wrongdoing.^45^ It is likely that one of the hospital employees was an informer and reported details of the Polish-American cooperation to the Public Security Office. The officers were perennially suspicious enough to investigate even the doctors’ joint outings to the cinema or to restaurants.^46^

The situation of the hospital in Piekary Śląskie was not an isolated case of medical mission entanglement in politics; it was, in fact, a mirror image of the problems faced by another flagship institution supported by the USC – the Varsovie Hospital in Toulouse. However, while in Poland the American mission was treated by the communist authorities as a potential outpost of Western intelligence, in France the vector of suspicion was reversed. The Varsovie Hospital, dedicated to refugees from the Spanish Civil War and run in cooperation with the Joint Anti-Fascist Refugee Committee (JAFRC), became a target of attacks within the United States itself during the era of rising McCarthyism. As a result of the campaign led by the House Committee on Un-American Activities (HUAC), which condemned cooperation with leftist organisations, the USC was forced to withdraw its support for the Toulouse facility in February 1948 to protect itself from accusations of supporting communism.^47^

The fates of both institutions perfectly illustrate the ‘double bind’ of Cold War humanitarian aid. In Piekary Śląskie, USC personnel were tolerated but subjected to strict surveillance by the Security Office (Urząd Bezpieczeństwa) out of fear of ‘imperialist influences’ and espionage. Conversely, the Varsovie Hospital, paradoxically, after losing American funding, fell victim to internal Stalinist purges, during which staff were accused of ‘Titoism’ and nationalist deviations.^48^ This history demonstrates that in the realities of the early Cold War, medical neutrality became a fiction: the USC in Poland had to manoeuvre to maintain its mission despite the reluctance of communist authorities, while in France it was forced to abandon it due to anti-communist hysteria in its own country.

The USC was regarded positively in Poland. High-ranking government officials considered the USC one of the finest relief agencies in Poland, stressing that it ‘sticks to its job, does a great deal of work and does not mingle in politics’. The Polish government also appreciated the USC’s work with Polish displaced persons in Germany, the establishment of the hospital at Piekary Śląskie, and the dispatch of teaching missions. The friendly attitude of the Polish government contributed to favourable publicity for the USC’s missions in Poland.

At the same time, in the Polish press aimed at citizens, Polish-American medical cooperation was used for information and propaganda purposes. As early as 1946, the local press was still boasting about the supply of penicillin for the miners provided by USC.^49^ ‘As part of the agreement, the hospital (in Piekary Śląskie) receives those medicines from America that we do not have at home’ – read one article written in 1947 in the Polish daily newspaper *Dziennik Zachodni* (‘Western Daily’).^50^ In 1948 *Gazeta Robotnicza* (‘Workers’ Newspaper’) wrote about ‘cooperation between foreign doctors and the Polish medical world’,^51^ and in the same year later continued the narrative of near-objectivity.^52^ The opening of the hospital in Piekary Śląskie was also a source of pride.^53^ In a newspaper that was the organ of the Polish Socialist Party, an article entitled ‘The Work of a Good Heart’ appeared in 1948. It was important because the author of the text explicitly called the American women responsible for organising the hospital ‘people of good will’ and ‘friends’.^54^ Likewise in 1950, *Dziennik Zachodni* published a letter written by parents of triplets. They thanked USC and Dorothea B. Jones (one of the ‘American friends’) for their help.^55^ Information on the USC’s charitable activities also appeared in the Polish press in the context of other assistance activities, for example in the aftermath of a flood (probably one which occurred in 1947).^56^ On the occasion of the arrival of the Americans in Poznań, where they gave lectures, the local press took great joy in reminding its readers of the words of one of the doctors that ‘they will never forget Poland’.^57^

While these papers reported on the USC mission, they did not merely reflect the facts; they actively reshaped them to serve political ends. This should come as no surprise, since from 1946 onwards all publications were subject to control by the Główny Urząd Kontroli Prasy, Publikacji i Widowisk (Main Office for the Control of the Press, Publications and Performances). The *Dziennik Zachodni* wrote about reciprocity in sharing knowledge and the American delegation learning of Polish achievements,^58^ as did *Głos Wielkopolski* (‘The Voice of Greater Poland’).^59^ One of the clearest examples of this narrative is an article of July 24, 1948, under the telling headline ‘American doctors’ mission to help Poland admires achievements of Polish medics’. It related how: ‘The nature of this visit does not bear the characteristics of furthering the training of our doctors or professors, but rather it is about exchanging mutual experiences, and establishing contact and helping to organise our facilities’.^60^ The press narrative was only partial because it deliberately downplayed the pedagogical and relief nature of the mission. The USC’s primary goal was to bring Polish medicine back to global standards after years of wartime isolation. By portraying it only as a ‘mutual exchange’, the press obscured the fact that Poland was a desperate recipient of aid and that the USC was providing training that the domestic system could not yet offer.

What is important is that press did not merely reflect state policy. It translated it into a socially acceptable form. By emphasising the friendship of American doctors, the press was not just producing propaganda, but managing a potential ‘ideological contamination’ risk. They isolated the medical aid from the American state to prevent the public from viewing the U.S. government as a saviour. The USC’s self-definition as an ‘apolitical’ entity was not merely a mission statement; it functioned as a vital diplomatic currency. Probably, this apolitical label was explicitly adopted by Polish press and authorised by the Main Office for the Control of the Press, Publications and Performances censors to create a safe rhetorical distance between the much-needed medical expertise and the ‘hostile’ Truman Doctrine after 1947. By framing the mission as a peer-to-peer scientific exchange, the authorities could utilise American know-how without granting the US government the political credit for Poland’s medical reconstruction.

The narrative described is extremely interesting, especially when juxtaposed with other newspaper articles from those years. For the purposes of this research, I have limited myself to just one newspaper title: *Głos Wielkopolski* which was previously cited. The newspaper ironically described the American aid provided to Italy, and pointing out its self-interestedness,^61^ the mission in Egypt was also criticised for being military in nature.^62^ Readers were informed that the Americans ‘violently want to incorporate’ the French zone into the American–British occupation zone of Germany (the so-called Bizonia, established in 1947 through the economic merger of the U.S. and British zones).^63^ There were even caricatures printed depicting America as the wolf from Little Red Riding Hood who wants to ‘devour’ France.^64^ Hundreds of such examples can be provided. Why, then, did the press praise USC’s assistance while simultaneously criticising the US and the assistance it offered?

The dual narrative – praising USC’s assistance while criticising the US – can be understood through the lens of communist propaganda’s strategic needs. Poland, devastated by war, required international aid for reconstruction, and the USC’s contributions, such as penicillin and hospital repairs, were tangible benefits that could be showcased to the public. Praising these efforts allowed the authorities to demonstrate openness to humanitarian aid while maintaining legitimacy among citizens facing postwar hardships.

Simultaneously, criticising broader US aid efforts served to reinforce anti-Western sentiment, aligning with the ideological stance of the communist regime. This was particularly important given Poland’s position as a Soviet satellite, where maintaining loyalty to the USSR and resisting alignment with the orbit of the USA was paramount. The press’s use of caricatures and ironic descriptions of American aid to other countries, such as Italy and Egypt, was part of a broader campaign to depict the US as an imperialist power, contrasting with the seemingly altruistic USC assistance.

A clearer chronology reveals that the propaganda discourse surrounding American aid was not a linear evolution from gratitude to hostility, but rather a complex management of contradictions. As early as 1945, the Polish press was permeated by a strong anti-Western narrative, depicting the United States as an imperialist power in the emerging geopolitical conflict. However, within this generally hostile ideological landscape, the coverage of the USC in 1946–1947 functioned as a pragmatic enclave. While regional papers like *Głos Wielkopolski* ridiculed US foreign policy (e.g., in Italy or Egypt) through ironic articles and caricatures, they simultaneously maintained a bubble of appreciative reporting regarding specific medical deliveries, such as penicillin for miners, frankly admitting domestic shortages.

One can hypothesise that the existence of this positive ‘enclave’ within an otherwise hostile propaganda landscape was largely enabled by the USC’s conscious strategy of self-presentation. By steadfastly defining itself as a non-governmental and apolitical entity, the Committee provided the communist authorities with a convenient rhetorical device. As indicated in its programmatic documents, the USC sought to insulate its mission from the ‘complexities of international politics’, which effectively allowed the regime’s press to construct a useful dichotomy: distinguishing between the ‘hostile’ imperialist U.S. government and ‘good’, ordinary Americans described as ‘people of good will’. This distinct non-state identity likely functioned as a protective shield. It permitted censors to authorize laudatory articles about American physicians because such praise did not contradict the broader anti-White House narrative; viewed through this lens, USC aid was not a ‘political weapon’ of the West, but rather evidence that progressive forces capable of cross-border cooperation existed even within a capitalist society.

‘It should be emphasised’, said one report, written in 1948, ‘that, in spite of the considerable number of different nationals working with the Mission, no discordant political note whatsoever disturbed the perfectly harmonious atmosphere in which the Mission conducted its work’.^65^ The local American press wrote: ‘The present Polish government, despite its Communist complexion, is fully aware of the friendly regard Americans have for the Poles. More than that, it is doing something to maintain that friendship’.^66^ The mission’s efforts to avoid political friction were likely a strategic necessity, given the rising tensions between East and West and the increasing suspicion with which foreign organisations were viewed by Communist authorities. These complementary perspectives: one internal and apolitical, the other external and cautiously diplomatic, offer valuable insight into how the mission was both perceived and managed in a highly politicised context. As such, they inform this article’s broader analysis of the USC’s work as a unique example of humanitarian diplomacy, navigating ideological boundaries while delivering tangible aid.

## Conclusions

This study has shown that, although modest in scale compared with larger relief agencies, the USC’s medical mission in Poland played a distinct and enduring role in the country’s postwar public‐health recovery. By tracing the Committee’s efforts, from the 1946 Medical Teaching Mission and the foundation of the Piekary Śląskie hospital team to targeted tuberculosis control and physicians’ training, we have documented how a small, faith‐based NGO combined direct care with professional education in a context marked by material scarcity and infrastructural collapse. The USC’s donations of textbooks, X‑ray machines, DDT for epidemic control, and thousands of hours of lectures helped to replenish not only supplies but also technical knowledge at a time when Polish medical schools and hospitals were operating at a fraction of their capacity.

Historiographically, the USC case challenges the monolithic narrative of the ‘frozen’ East-West divide. Aligned with Sandrine Kott’s perspective on international organisations, this mission reveals that the Iron Curtain was not a hermetic seal but rather a porous membrane through which knowledge and expertise continued to circulate.^67^ Scholars such as György Péteri have proposed the metaphor of a ‘Nylon Curtain’ to describe the complex, often transparent nature of the divide, arguing that cultural and intellectual exchanges persisted despite political freezing.^68^ Using his analysis, it can be assumed that American doctors in Piekary Śląskie were precisely the element that penetrated the ‘Nylon Curtain’, providing know-how and modern medical standards. The USC did not merely deliver aid; it fostered a transnational epistemic community. By facilitating direct, peer-to-peer exchanges between American and Polish physicians during the postwar period, the Committee maintained a space for professional dialogue that operated beneath the radar of high-level diplomatic paralysis. This suggests that the history of postwar humanitarianism should not be written solely through the lens of state actors and superpowers, but must account for the agility of smaller non-governmental actors capable of navigating geopolitical fissures that blocked larger institutions.

At the same time, analysis has underscored the extent to which humanitarian relief in the emerging Cold War was inseparable from ideological contestation. USC personnel in Piekary Śląskie and elsewhere found themselves under constant surveillance; their work was alternately celebrated in domestic newspapers as evidence of American ‘good will’ and denounced in parallel propaganda campaigns aiming to cast US aid as a tool of Western political influence. These tensions highlighted a broader paradox of postwar assistance: even the most ostensibly neutral medical interventions became arenas for competing narratives about sovereignty, loyalty, and the rightful beneficiaries of reconstruction. In so doing, it revealed the limits of quantitative measures of assistance (pounds of supplies, numbers of lectures) when set against the more elusive question of lasting impact on knowledge, professional practice, and public trust. As Józef Łaptos observed, we can enumerate the USC’s inputs, but ‘we cannot answer the question to what extent they stimulated the deepening of knowledge in order to help others, what horizons they opened in the minds of the listeners’.^69^

Future research might build on this study by examining the subsequent careers of Polish physicians who participated in USC‐sponsored training, or by comparing the Committee’s interventions with those of other small NGOs operating in Soviet‑dominated Europe. Such work could help clarify whether the pedagogical methods introduced by the USC – particularly its emphasis on peer‐to‐peer exchange and on integrating clinical teaching into everyday hospital practice – found lasting purchase in Polish medical pedagogy. Ultimately, the USC’s mission in Poland reminds us that postwar reconstruction was not only a matter of material transfer but also of intellectual exchange, and that even in the shadow of great‐power rivalry, spaces sometimes existed for cross‐border solidarity grounded in professional and humanitarian values.

## Footnotes

1 Jon Arrizabalaga, Àlvar Martínez-Vidal, ‘Unitarian Service Committee’s activities with refugee populations and the Resistance in France during and after the Second World War’, in Marie-Luce Desgrandchamps, Laure Humbert, Bertrand Taithe and Raphaële Balu (eds.), *Medical Care, Humanitarianism and Intimacy in the Long Second World War, 1931–1953* (Manchester: Manchester University Press, 2025), 178.

2
*Ibid*.

3 Andover-Harvard Theological Library, Harvard Divinity School (AHTL), bMS 16103, Box 39, Folder 8, The USC Program in France, 1.

4
*Ibid.*

5 AHTL, bMS 16103, Box 29, Folder 1, Organizational Diagram of the Medical Mission to Poland and Finland.

6 AHTL, bMS 16103, Box 40, Folder 5, (Programs and Projects) Schedule B, Section II – National, 2.

7 AHTL, bMS 16103, Box 39, Folder 6, USC Medical Missions Demonstrate Democracy in Action, 5.

8

Olga Bogatyreva , ‘Humanitarian Diplomacy: Modern Concepts and Approaches’, Herald of the Russian Academy of Sciences, 92, Suppl 14 (2022), 1350–52.

9

Chester M. Jones , ‘Medical Mission to Greece and Italy’, The New England Journal of Medicine, 240, 14 (1949), 529–32.10.1056/NEJM19490407240140118115427

10

Susan Subak and William F. Schultz , Rescue and Flight: American Relief Workers Who Defied the Nazis (Lincoln: University of Nebraska Press, 2010).

11

Haim Genizi , ‘Christian charity: the Unitarian Service Committee’s relief activities on behalf of refugees from Nazism, 1940–5’, Holocaust and Genocide Studies, 2, 2 (1987), 261–76.10.1093/hgs/2.2.26120684119

12

Aurelio Velázquez-Hernández , ‘The Unitarian’s Service Committee Marseille Office and the American networks to aid Spanish refugees. (1940–1943)’, Culture & History Digital Journal, 8, 2 (2019), 1–10.

13 Jon Arrizabalaga, ‘La asistencia médica a los refugiados en Francia durante la Segunda Guerra Mundial: la Clínica de Marsella (1941–1945)’, *Dynamis*, 40, 1 (2020), 67–91; Jon Arrizabalaga and Àlvar Martínez-Vidal, ‘Medicine, Religion, and the Humanitarian Ethos: Walter B. Cannon, Unitarianism, and the Care of Spanish Republican Refugees in France’, *Journal of the History of Medicine and Allied Sciences*, 77, 2 (2022), 158–85; Arrizabalaga, Martínez-Vidal, *op. cit.* (note 1), 178–201.

14

Shigemasa Ikeda , ‘The Unitarian Service Committee Medical Mission: Contribution by the United States to Post-World War II Japanese Anesthesiology’, Anesthesiology, 106, 1 (2007), 178–8510.1097/00000542-200701000-0002717197860; Shigemasa Ikeda , ‘American Anesthesiologists’ Contribution to Post-World War II Global Anesthesiology: the Unitarian Service Committee’s Medical Missions’, Journal of Clinical Anesthesia, 23, 3 (2011), 244–52.10.1016/j.jclinane.2010.08.01321570621

15

Akitomo Matsuki , ‘Influence of the Unitarian Service Committee Medical Mission on the Development of Anesthesiology in Japan – Focusing on the Influence of Saklad’s Lectures of the First Mission in 1950’ Masui, The Japanese Journal of Anesthesiology, 65, 8 (2016), 858–62.30351604

16

Miklós Dániel , ‘Egy online gyűjtemény magyar vonatkozásai. Magyar relevancia a Unitarian Service Committee online elérhető anyagaiban’, Levéltári Szemle, 74, 2 (2024), 51–8.

17

Hans Herken , Die Berliner Pharmakologie in der Nachkriegszeit Erinnerungen an ein Stück bewegter Universitätsgeschichte der Jahre 1945–1960 (Berlin-Heildelberg: Springer, 1999).

18 Jessica Reinisch, ‘“We Shall Rebuild Anew a Powerful Nation”: UNRRA, Internationalism and National Reconstruction in Poland’, *Journal of Contemporary History*, 43, 3 (2008), 451–76; Sławomir Łotysz, ‘A “Lasting Memorial” to the UNRRA? Implementation of the Penicillin Plant Programme in Poland, 1946–1949’, *Icon*, 20, 2 (2014), 70–91; Sławomir Łotysz, ‘国际卫生组织与冷战初期盘尼西林 生产方式的传播 – 以联合国善后救济总署在欧活动和美国 医药援华会在华事务为例’, *医疗社会史研究*, 2, 1 (2017), 3–31; Sławomir Łotysz, ‘Penicylina i protezy. Pomoc UNRRA w odbudowie polskiego przemysłu medycznego po II wojnie światowej’, *Kultura Współczesna: teoria, interpretacje, Krytyka*, 102, 3 (2018), 118–30; Józef Łaptos , Humanitaryzm i polityka . Pomoc UNRRA dla Polski i polskich uchodźców w latach 1944–1947 (Kraków: Wydawnictwo Naukowe Uniwersytetu Pedagogicznego, 2018); Sławomir Łotysz, ‘Kraków czy Tarchomin? Historia sporu o lokalizację fabryki penicyliny przekazanej Polsce w ramach programu pomocowego UNRRA (1946–1947)’, *Analecta: Studia i Materiały z Dziejów Nauki*, 23, 1 (2018), 289–310; Sławomir Łotysz, *Fabryka z darów. Penicylina za żelazną kurtyną 1945*–*1954* (Warszawa: Oficyna Wydawnicza ASPRA-JR, 2020); Yulia Zapariy and Ksenia Borodunina, ‘UNRRA Mission in Poland 1945–1947: Successes and Failures of the International Humanitarian Effort’, *Izvestiya of Samara Scientific Center of the Russian Academy of Sciences History Sciences,* 4, 4 (2022), 94–100.

19 György Péteri, ‘Nylon Curtain – Transnational and Transsystemic Tendencies in the Cultural Life Of State-Socialist Russia Aand East-Central Europe’, *Slavonica,* 10, 2 (2004), 113–23.

20 See: *Dzieje medycyny w Polsce. Opracowania i szkice. Tom 3 – Lata 1944–1989*, red. Wojciech Noszczyk (Warszawa: Państwowy Zakład Wydawnictw Lekarskich, 2016).

21 AHTL, bMS 16103, Box 39, Folder 8, Copy. Unitarian Service Committee International Hdqs. Report, 1.

22
*Ibid*, 1–2.

23 AHTL, bMS 16103, Box 29, Folder 18, 5–7.

24 AHTL, bMS 16103, Box 29, Folder 18, 8–9.

25 AHTL, bMS 16103, Box 29, Folder 18, 2–31.

26 AHTL, bMS 16103, Box 29, Folder 18, 93–102. See also (with a slight difference in some data): Łaptos, *op. cit.* (note 18), 491.

27 AHTL, bMS 16103, Box 39, Folder 8, Outline of Polish Relief by Unitarian Service Committee, April 1947, 1–4.

28 AHTL, bMS 16103, Box 29, Folder 16, Report sent by the Polish Medical Mission from Mauvezin, 1 October 1945, 3.

29 AHTL, bMS 16103, Box 29, Folder 16, Report on the Work of the Medico-Social Mission during the Repatriation of Polish Deportees from France, 10 December 1945, 1.

30 AHTL, bMS 16103, Box 29, Folder 16, Report on the Work of the Medico-Social Mission during the Repatriation of Polish Deportees from France, 10 December 1945, 3.

31 AHTL, bMS 16103, Box 39, Folder 8, Outline of Polish Relief by Unitarian Service Committee, April 1947, 1–4.

32 AHTL, bMS 16103, Box 29, Folder 19, Diary of Dr. Wade Oliver, July–August 1946, 25.

33
*Ibid*, 19.

34 AHTL, bMS 16103, Box 39, Folder 8, Outline of Polish Relief by Unitarian Service Committee, April 1947, 1–4.

35 AHTL, bMS 16103, Box 39, Folder 8, Outline of Polish Relief by Unitarian Service Committee, April 1947, 1–4.

36 Elżbieta Więckowska, *Zwalczanie ostrych chorób zakaźnych w Polsce w latach 1944–1950* (Wrocław: Wydawnictwo Uniwersytetu Wrocławskiego, 2001).

37 AHTL, bMS 16103, Box 30, Folder 13, Contract between Unitarian Service Committee of Boston, Massachusetts, USC and Zakład Ubezpieczeń Społecznych, Warsaw, Poland, 2.

38 See: Kacper Rosner-Leszczyński, ‘X-ray Wagons in the Fight against Tuberculosis after World War II: Primary Source Analysis and Presentation of Chosen Iconography’, *Epidemiological Review,* 78, 4 (2024), 414–27.

39 State Archive in Wrocław, Urząd Wojewódzki Wrocławski, Wydział Zdrowia, XVI/184, Zarządzenia, okólniki, statuty i korespondencja, 46.

40 AHTL, bMS 16103, Box 35, Folder 3, Medical mission to Poland and Finland, July 1–August 27, 1948, published report, 10, 77, 79.

41 AHTL, bMS 16103, Box 39, Folder 8, Outline of Polish Relief by Unitarian Service Committee, April 1947, 1–4.

42 Sandrine Kott, ‘Par-delà la guerre froide. Les organisations internationales et les circulations Est-Ouest (1947–1973)’, *Vingtième Siècle. Revue d’histoire,* 109 (2011), 153.

43 Kornelia Banaś, ‘Amerykański szpital i jego podejrzana kadra. Z historii piekarskiej „urazówki”’, *CzasyPismo. O historii Górnego Śląska,* 1 (2017), 63.

44
*Ibid.*

45 AHTL, bMS 16103, Box 32, Folder 4, Final Report Phase II, Kosciuszko Hospital, Piekary, 3.

46 Banaś, *op. cit.* (note 43), 64.

47 Arrizabalaga, Martínez-Vidal, *op. cit.* (note 1), 189–90.

48
*Ibid.*

49 ‘Penicylina dla górników’, *Słowo Polskie*, 1, 24 (1946), 4.

50 (k), ‘Nowy Szpital w Piekarach’, *Dziennik Zachodni Wieczór*, 3, 47 (1948), 2.

51 (km), ‘Współpraca lekarzy zagranicznych z polskim światem lekarskim’, *Gazeta Robotnicza,* 57, 206 (1948), 5.

52 H, ‘Instytut Chirurgii Urazowej w Piekarach Śląskich dobrodziejstwem robotników’, *Gazeta Robotnicza,* 57, 51 (1948), 7.

53 ‘Dr A. Powell Davies na otwarciu szpitala w Piekarach Śląskich’, *Głos Ludu. Pismo Polskiej Partii Robotniczej,* 5, 54 (1948), 2.

54 ‘Dzieło dobrego serca’, *Robotnik. Centralny organ PPS,* 54, 11 (1948), 6.

55 ‘Rodzice trojaczków dziękują’, *Dziennik Zachodni,* 6, 8 (1950), 5.

56 ‘Towarzystwa charytatywne spieszą z pomocą powodzianom’, *Robotnik. Centralny organ PPS*, 54, 172 (1948), 3.

57 ‘Ekipa lekarzy zagranicznych opuściła Poznań’, *Głos Wielkopolski*, 4, 221 (1948), 2.

58 ‘Międzynarodowa misja lekarzy’, *Dziennik Zachodni*, 4, 207 (1948), 2.

59 ‘3 sierpnia przybędzie do Poznania misja amerykańska’, *Głos Wielkopolski*, 4, 200 (1948), 2.

60 ‘Misja amerykańskich lekarzy dla niesienia pomocy Polsce podziwia osiągnięcia polskiej medycyny’, *Głos Wielkopolski*, 4, 201 (1948), 2.

61 ‘Amerykańska miłość’, *Głos Wielkopolski*, 4, 103 (1948), 3.

62 ‘Niezwykła „misja medyczna” w Egipcie’, *Głos Wielkopolski*, 4, 106 (1948), 2.

63 ‘Amerykanie przemocą włączyć chcą strefę francuską do Bizonii’, *Głos Wielkopolski*, 4, 231 (1948), 1.

64 ‘Francuski „czerwony kapturek”’, *Głos Wielkopolski*, 4, 123 (1948), 2.

65 AHTL, bMS 16103, Box 35, Folder 3, Medical mission to Poland and Finland, July 1– August 27, 1948, published report, 7.

66 ‘Americans About Poland’, *Poland of Today*, 4 (1948), 20.

67 Kott, *op. cit.* (note 42), 143–54.

68 Péteri, *op. cit.* (note 19), 113–23.

69 Łaptos, *op. cit.* (note 18), 438. In original: ‘Można zestawiać liczby tych gości, godziny wykładów, pokazów czy instruktażów, ale – podobnie jak w wypadku współczesnych akcji humanitarnych – nie potrafimy odpowiedzieć na pytanie, w jakim stopniu stymulowały one do pogłębiania wiedzy w celu niesienia pomocy innym, jakie horyzonty otwierały w umysłach słuchaczy’.

## Long descriptions

### Figure 1. Long description

Line graph titled ’Comparison of aid from 3 selected organisations’ showing financial aid to Poland in USD from 1946 to 1949 for three organisations. The American Joint Distribution Committee (AJDC) provided the most aid, starting at approximately 3,600,000 in 1946 and declining steeply each year to around 200,000 by 1949. The Cooperative for American Remittances to Europe (CARE) contributed moderately, remaining relatively stable between 550,000 and 700,000 from 1946 to 1948 before dropping to roughly 200,000 in 1949. The Unitarian Service Committee (USC) provided the smallest amounts, staying near the baseline throughout the period, rising slightly from about 30,000 in 1946 to around 70,000 in 1948, then declining by 1949. All three organisations show a convergent downward trend by 1949.

Navigate back to Figure 1..

